# Gab2 mediates hepatocellular carcinogenesis by integrating multiple signaling pathways

**DOI:** 10.1096/fj.201700120RR

**Published:** 2017-08-21

**Authors:** Jianghong Cheng, Yanhong Zhong, Shuai Chen, Yan Sun, Lantang Huang, Yujia Kang, Baozhen Chen, Gang Chen, Fengli Wang, Yingpu Tian, Wenjie Liu, Gen-Sheng Feng, Zhongxian Lu

**Affiliations:** * School of Pharmaceutical Sciences, State Key Laboratory of Cellular Stress Biology, Xiamen University, Xiamen, China;; † Department of Pathology, Fujian Provincial Tumor Hospital, Fuzhou, China;; ‡ Division of Biological Sciences, Department of Pathology, University of California, San Diego, La Jolla, California, USA

**Keywords:** HCC, knockout mouse, IL-6, Jak2/Stat3 signaling pathway, therapeutic target

## Abstract

Our previous studies have found that Growth factor receptor-bound protein 2–associated binding protein 2 (Gab2)—a docking protein—governs the development of fatty liver disease. Here, we further demonstrate that Gab2 mediates hepatocarcinogenesis. Compared with a faint expression in *para*-carcinoma tissue, Gab2 was highly expressed in ∼60–70% of human hepatocellular carcinoma (HCC) specimens. Deletion of Gab2 dramatically suppressed diethylnitrosamine-induced HCC in mice. The oncogenic effects of Gab2 in HepG2 cells were promoted by Gab2 overexpression but were rescued by Gab2 knockdown. Furthermore, Gab2 knockout in HepG2 cells restrained cell proliferation, migration and tumor growth in nude mice. Signaling pathway analysis with protein kinase inhibitors demonstrated that oncogenic regulation by Gab2 in hepatic cells involved multiple signaling molecules, including ERK, Akt, and Janus kinases (Jaks), especially those that mediate inflammatory signaling. IL-6 signaling was increased by Gab2 overexpression and impaired by Gab2 deletion *via* regulation of Jak2 and signal transducer and activator of transcription 3 phosphorylation and the expression of downstream genes, such as *Bcl-2* (B-cell lymphoma 2), *c-Myc*, *MMP7* (matrix metalloproteinase-7), and *cyclin D1* *in vitro* and *in vivo*. These data indicate that Gab2 mediates the pathologic progression of HCC by integrating multiple signaling pathways and suggest that Gab2 might be a powerful therapeutic target for HCC.—Cheng, J., Zhong, Y., Chen, S., Sun, Y., Huang, L., Kang, Y., Chen, B., Chen, G., Wang, F., Tian, Y., Liu, W., Feng, G.-S., Lu, Z. Gab2 mediates hepatocellular carcinogenesis by integrating multiple signaling pathways.

Hepatocellular carcinoma (HCC) is one of the most common types of cancer worldwide and the third leading cause of cancer mortality (1). According to the latest epidemiologic survey, ∼782,500 new cases of liver cancer and 745,500 liver cancer–related deaths occurred worldwide in 2012, with half of these patients and deaths in China alone (2). Unfortunately, effective prevention and treatment strategies for HCC are limited, and almost all trials of molecular targeted therapy have experienced failure as a result of the complex molecular mechanism that underlies HCC development and progression.

Only a small fraction of HCC cases are attributed to gene mutation—the majority of cases develop in response to chronic injuries and liver diseases, such as chronic liver cirrhosis, hepatitis B virus (HBV) or hepatitis C virus, alcohol consumption, nonalcoholic steatohepatitis, and food contamination (3, 4). A recent study of the average percentage contribution of HCC-related etiologies to cases in Europe and America reported that glucose and lipid metabolism disorders account for 36.6%, alcoholic disorder for 23.5%, hepatitis C virus for 22.4%, HBV for 6.3%, and other genetic mutations for 3.2% (5). Hepatocellular carcinogenesis involves a variety of pathologic factors, including viruses, growth factors, metabolic molecules, and inflammatory cytokines. These pathologic factors can trigger a series of signaling pathways that can lead to HCC formation, invasion, and metastasis (6–8).

The oncogene *HBx*, which encodes the HBV X protein, can integrate the Ras/Raf/MAPK, MEKK1/JNK, and PI3K/Akt/mammalian target of rapamycin signaling pathways, all of which are involved in cell proliferation and survival (9). Abnormal activation of Wnt/β-catenin signaling also commonly occurs in HCC, and this pathway can regulate the expression of key proliferation-related genes, such as cyclin D (10). Excessive alcohol consumption disturbs glycolipid metabolism and results in excessive fat accumulation, which can stimulate an inflammatory response, create oxidative stress, and further damage hepatocytes. These pathologic processes recruit TNF-α, IL-6, peroxisome proliferator-activated receptor-α/retinoid X receptor, 5′−AMPK/acetyl-CoA carboxylase, NAD-dependent deacetylase sirtuin-1/sterol regulatory element-binding transcription factor 1, PI3K/Akt–sterol regulatory element-binding transcription factor 1, JAK–signal transducer and activator of transcription 3 (Stat3), and other signaling molecules to promote the development of HCC (11). Furthermore, Notch (12), IGF/IGF receptor (13), hepatocyte growth factor/hepatocyte growth factor receptor (14), and EGFR (15) are also involved in hepatocellular carcinogenesis. These signaling molecules form an intricate network that governs carcinogenesis, and hepatocarcinogenesis is the result of various integrated signals. As a result of the complexity of hepatocellular carcinogenesis, it is improbable that an effective therapeutic target will arise from a single signaling pathway; therefore, it is necessary to identify a hub protein that can integrate multiple signaling pathways—similar to EGFR and 14-3-3 proteins—to develop a new, effective drug for the treatment of liver cancer.

Growth factor receptor-bound protein 2–associated binding protein 2 (Gab2), a scaffolding protein, can integrate and amplify several upstream receptor signals and recruit various intracellular downstream signaling proteins, including Ras (16), proto-oncogene tyrosine-protein kinase Src (17), protein tyrosine phosphatase SHP2 (18), PI3K (19), adapter molecule Crk (20), and PLC (21). Our previous study demonstrated that Gab2 recruited PI3K/Akt, suppressor of cytokine signaling 3 (Socs3)/Stat3, and other signaling molecules in fatty liver induced by pathologic factors. These pathways also play critical roles in regulating cell proliferation and survival (22). Furthermore, Gab2 governs tumorigenic signaling and is a novel potential therapeutic target in leukemia, breast cancer, ovarian cancer, and melanoma (23); therefore, we hypothesized that Gab2 may be involved in hepatocellular carcinogenesis.

To verify our hypothesis, we investigated Gab2 protein levels in human HCC and determined the function of Gab2 in hepatocellular carcinogenesis by using transgenic mice. Our results demonstrate that Gab2 is overexpressed in HCC and promotes the formation of liver cancer by integrating several signaling pathways, including the ERK, Akt, and Janus kinase (Jak) pathways. These results suggest an oncogenic role for Gab2 in hepatocellular carcinogenesis.

## MATERIALS AND METHODS

### Fresh HCC samples and tissue microarray

Fresh human HCC and paracarcinoma tissues were collected from the Department of Pathology, Fujian Provincial Tumor Hospital, between March 2013 and November 2014. The study protocol was approved by the Research Ethics Committee of Fujian Provincial Tumor Hospital (201515), and written informed consent was obtained from all participants. Paracarcinoma tissues were collected at a distance of at least 2 cm from tumor lesions. Tumor and paracarcinoma tissues were fragmented into 0.1-cm^3^ pieces and stored at −80°C. Protein extracts from tissues were stored at −80°C for Western blot analysis.

A tissue microarray that contained 23 cases of normal liver tissue and 94 cases of HCC tissue was purchased from Alenabio Company (Xi’an, China). Gab2 expression was assayed by immunohistochemistry (IHC) with a Gab2 Ab (06-967; Millipore, Billerica, MA, USA) and comprehensively evaluated according to staining intensity and the number of positive cells.

### Diethylnitrosamine-induced primary liver cancer

C57BL/6 mice were obtained from the Laboratory Animal Center of Xiamen University. *Gab2* transgenic mice were a gift from the Burnham Institute for Medical Research (San Diego, CA, USA) (24). Mice were housed under standard conditions with free access to food and water. All experimental procedures were approved by the Animal Welfare Committee of Research Organization (X201011), Xiamen University. Mice were housed 3–5 per cage in a room under controlled light (12 h/d) and temperature (22 ± 2°C) conditions. For chemical-induced hepatocarcinogenesis, mice at postnatal day 15 received an intraperitoneal injection of diethylnitrosamine (DEN; 25 mg/kg; N0258-1G; Sigma-Aldrich, St. Louis, MO, USA) or isometric saline. Then, mice were weaned and maintained on regular chow. Mice were euthanized at 6 and 12 mo after initial injection, and livers were harvested, stored, and photographed for the next analysis. Tumor and paracarcinoma tissue samples—tissues at a distance of 0.5 cm from tumor lesions were defined as paracarcinoma—were separated from the whole liver. Tissues were then divided into 0.5-cm squares for paraffin embedding and extraction of total protein and RNA.

### Xenograft model

For xenograft experiments, 4 different groups of cells (2 × 10^6^ cells per mouse) were s.c. injected into 5- to 6-wk-old nude mice (*n* = 8/group). After 6 d, tumor size was measured with Vernier calipers every 2 d. At the 20th day after subcutaneous injection, tumors were photographed using the Caliper IVIS Lumina II system (Caliper Life Sciences, Hopkinton, MA, USA) and then removed for the next experiments. Tumor volume was calculated as 0.5 × length × width^2^.

### Histochemistry, IHC, and ELISA

Histochemical staining (hematoxylin and eosin) and IHC were performed according to a previously published standard protocol (22). IHC analysis of tumor lesions using Ki-67 (4203-1; Epitomics, Burlingame, CA, USA), PH3 (9701; Cell Signaling Technology, Danvers, MA, USA), Gab2 (3239; Cell Signaling Technology), NF-κB (sc-8008; Santa Cruz Biotechnology, Santa Cruz, CA, USA), and CD68 (ab955; Abcam, Cambridge, United Kingdom) Abs was performed to detect the number of proliferating cells and/or inflammatory cells.

For ELISA, mouse blood was collected and incubated for 2 h at room temperature. Serum was acquired after centrifugation for 3 min at 9000 rpm, and TNF-α and IL-6 serum levels were measured by using ELISA kits according to manufacturer instructions (Blue Gene, Shanghai, China).

### Cell line construction and cell treatment

HepG2 human hepatoblastoma cell line was purchased from American Type Culture Collection (Manassas, VA, USA). To construct stable cell lines, a Gab2 cDNA fragment was inserted into pIRES2-EGFP (bicistronic Gab2-IRES-GFP expression cassette) or p3xFlag that contained a neomycin-resistance cassette (Neo^r^). All plasmids were transfected into HepG2 cells, and cells that stably express Gab2 were selected with G418. In brief, RNA interference–mediated knockdown was performed by transfecting Gab2–small interfering RNA (GGAGTGCCAGCTTCTCTCA) into HepG2 cells by using Lipofectamine 2000. To create the Gab2-knockout (KO) cell line, we built the Px459-gRNA plasmid to knock out endogenous Gab2 by using CRISPR technology and selected KO cells with puromycin. gRNA sequences are as follows: forward, CACCGCAGGTAAAAGGTGCGTTCAC; reverse, AAACGTGAACGCACCTTTTACCTGC. All cell lines were cultured at 37°C in a humidified incubator in 5% CO_2_ with DMEM that contained 10% fetal bovine serum (HyClone, Waltham, MA, USA). For cell treatments, indicated cells were treated with 50 ng/ml IL-6 (200-06-5 μg; PeproTech, Rocky Hill, NJ, USA) 24 h after seeding, then harvested at 0, 1, 3, or 6 h.

### MTT assay

Cells from different groups were seeded at an initial density of 2000 cells/well in 96-well plates and allowed to grow for 4 d, and cell number was assessed each day with an MTT assay [3-(4,5-dimethylthiazol-2-*yl*)-2,5-diphenyltetrazolium bromide; Sigma-Aldrich]. Absorbance was measured at 492 nm using a Multiscan plate reader (MK3; Thermo Fisher Scientific, Waltham, MA, USA).

### Migration assay

Cell migration was evaluated with wound healing and transwell assays. For wound healing experiments, a linear wound was created by using a pipette tip when cells reached 90% confluence. Cells were then washed twice with PBS and incubated in serum-free DMEM. Cell migration area was examined by using an inverted microscope and images were obtained at different time points. For transwell assays (PIEP12R48; Millipore), 1 × 10^5^ cells were seeded on a fibronectin-coated polycarbonate membrane and the membrane was placed into a transwell apparatus (Millipore). After incubation for 12–18 h, cells on the upper surface of the membrane were removed, and cells on the lower surface of the membrane were stained with 0.1% crystal violet. The stain was dissolved in 33% glacial acetic acid and quantitated with an ELISA plate reader at 492 nm, or the number of stained cells was counted under a microscope. All experiments were performed in triplicate.

### Apoptosis assay

Cells were seeded in 12-well plates and allowed to grow to 80% confluence after pretreating with 5-fluorouracil (100 μg/ml) for 36 h. Cells were then digested and washed twice with ice-cold PBS. After transient centrifugation for 5 min at 500 *g*, cells were incubated in darkness for 15 min in 100 μl ice-cold Annexin V binding buffer that contained 5 μl Annexin V-FITC and 5 μl propidium iodide. Eventually, the proportion of apoptotic cells was analyzed with flow cytometry (Millipore).

### Western blot analysis

Western blot assays were performed as previously described (22). Primary Abs against Gab2, P-Stat3^Tyr705^, Stat3, and Jak2 were purchased from Cell Signaling Technology. P-Jak2^Tyr1007/Tyr1008^ was obtained from Abcam. Western blot data are representative of 3 independent experiments. Analysis of grayscale intensity of Gab2 was proceeded by using Quantity 1 analysis software (Bio-Rad, Hercules, CA, USA). First, optimized images were chosen to create lanes, and band analysis was performed according to software manuals. Eventually, the grayscale of Gab2 was normalized to the grayscale of β-actin, and relative grayscale of Gab2 was analyzed by using GraphPad Prism 5.0 software (GraphPad Software, La Jolla, CA, USA).

### Quantitative RT-PCR

Total RNA was extracted with Trizol Reagent (Roche, Indianapolis, IN, USA) according to the manufacturer’s protocol and were reversed transcribed with an All-in-One First-Strand cDNA Synthesis Kit (Transgene, Beijing, China). Primer sequences used in the study are shown in Supplemental Table 1. Target gene mRNA levels were determined by quantitative PCR by using SYBR Green (Roche) and were normalized to glyceraldehyde 3-phosphate dehydrogenase mRNA levels. Relative gene expression was calculated by using the 2^−∆*Ct*^ method.

### Statistical analyses

All experiments were performed at least 3 times. Numerical data are presented as means ± sem, and these data were statistically analyzed by a 1-tailed Student’s *t* test and 1-way ANOVA by using GraphPad Prism 5.0 software. Statistically significant differences were accepted at *P* ≤ 0.05.

## RESULTS

### Gab2 is overexpressed in human HCC

**Figure 1.**
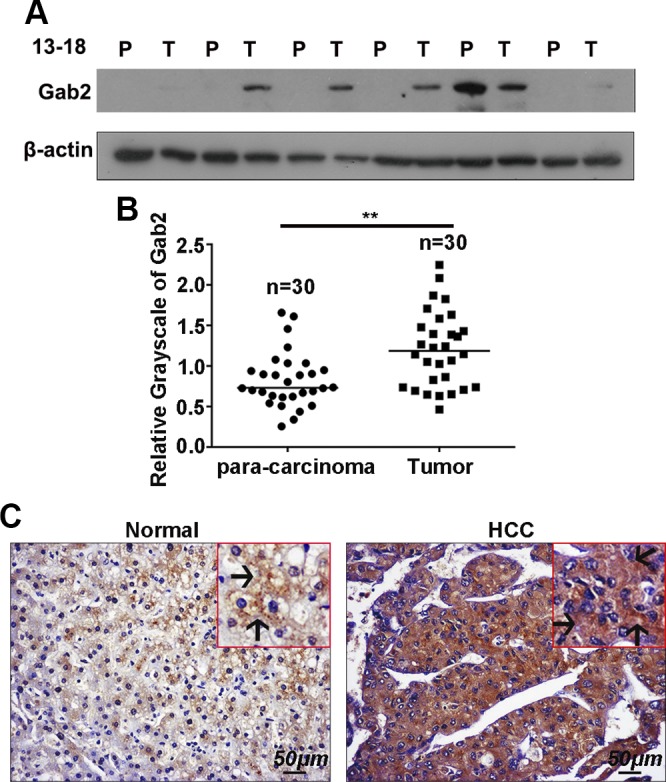
Gab2 is highly expressed in human HCC samples. *A*) Gab2 protein levels in 30 pairs of HCC tissue [tumor (T)] and adjacent nontumor tissue [paracarcinoma (P)] was determined by Western blotting. *B*) Grayscale quantitation of Gab2 protein expression. *C*) Representative IHC images of Gab2 protein expression in the array that contained HCC and normal liver tissue. Arrows indicate positive staining in the cytoplasm. Brown color indicates positive staining for Gab2. **P < 0.01.

To ascertain the role of Gab2 in liver carcinogenesis, Gab2 protein expression in human HCC was investigated. First, fresh tumor tissues and adjacent nontumor (paracarcinoma) tissues were collected from 30 cases of human liver carcinoma. Gab2 protein levels in these clinical specimens were detected by Western blotting. Gab2 expression was low in most of the paracarcinoma tissues, but was much higher in 70% of HCC samples (21 of 30; **Fig. 1*A*** and Supplemental Fig. 1). Quantitative analysis on the basis of the grayscale images of the bands indicated that Gab2 expression was significantly higher in HCC than in paracarcinoma tissue (Fig. 1*B*). Furthermore, Gab2 protein levels in tissue arrays that contained 94 HCC samples were assayed *via* IHC staining. Gab2 expression was high in most HCC samples (55/94; 58.5%), but in only a few normal liver tissue samples (6/23; 23%; Fig. 1*C* and **Table 1**). The significant difference in Gab2 expression between HCC and nontumor tissue indicates that Gab2 is involved in hepatic carcinogenesis.

**TABLE 1.** IHC staining of Gab2 in human HCC and normal liver tissue

Type	Positive expression (*n*)				Positive rate (%)
	+++	++	+	−	
Normal	0	4	2	17	26 (6/23)
HCC	9	23	23	39	58.5 (55/94)*

−, +, ++, and +++ represent different degrees of staining. *P < 0.05.

### Gab2 deletion suppresses DEN-induced liver tumorigenesis in mice

**Figure 2.**
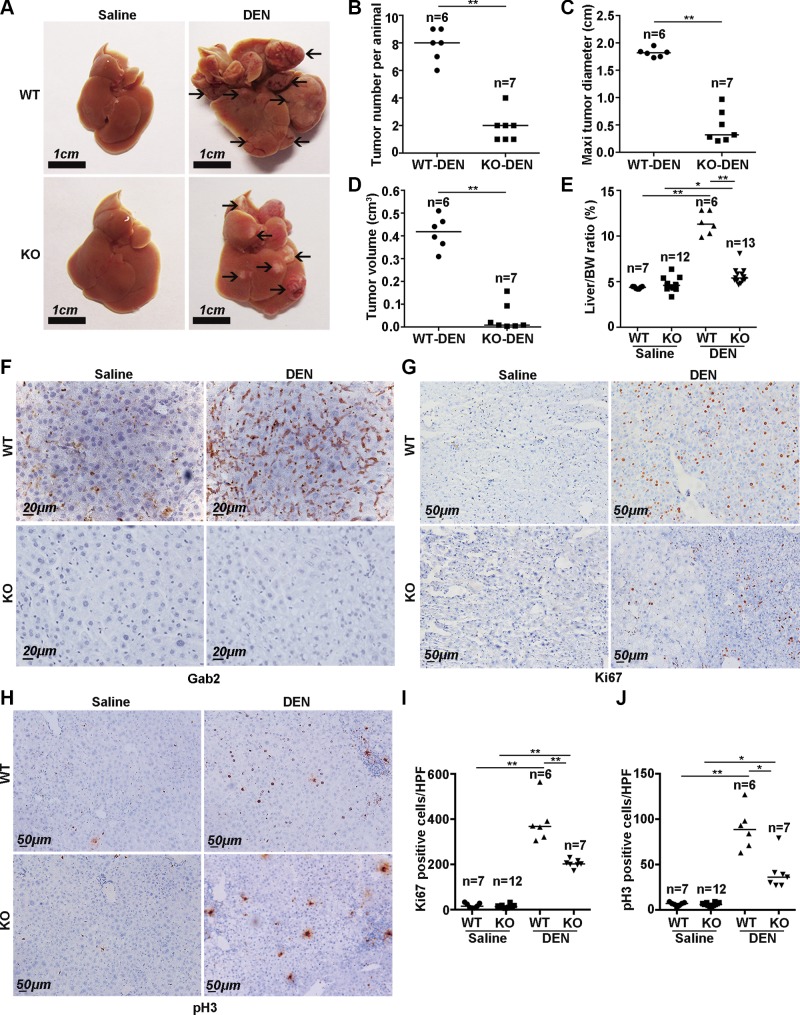
Gab2 ablation suppresses the development of DEN-induced primary liver cancer in mice. *A*) Characteristics of livers from mice that were treated with DEN for 12 mo. Arrows indicate liver cancer nodules. *B*–*E*) Tumor count per animal (*B*), average maximum tumor diameter (*C*), average tumor size (*D*), and average liver-to-body weight ratio (*E*) were compared between WT-saline, WT-DEN, Gab2-KO-saline, and Gab2-KO-DEN mice. *F*) Gab2 expressions of 4 group mouse models were shown with IHC staining. *G*, *I*) Representative IHC images of Ki-67 expression (*G*) and the number of Ki-67–positive cells (*I*) in liver tissue from DEN-treated mice [*n* = 5 high-power fields (HPFs) for each mouse]. *H*, *J*) pH3 expression by IHC (*H*) and statistical analysis of pH3-positive cells (*J*) in 4 groups of mice. Brown color indicates positive staining (*n* = 5 HPFs for each mouse). *P < 0.05, **P < 0.01.

To elucidate the role of Gab2 in hepatic carcinogenesis, we examined whether Gab2 affects liver tumor formation and progression. Gab2-KO and wild-type (WT) mice were i.p. injected with DEN. After 12 mo of DEN treatment, Gab2 levels were up-regulated significantly in WT mice compared with control mice with saline treatment, which suggests that Gab2 expression was possibly related to liver tumorigenesis (**Fig. 2*F***, upper). We then evaluated the tumorigenicity in DEN-evoked WT and Gab2-KO mice and found that there were many tumor nodules on the surface of livers from DEN-treated WT mice (Fig. 2*A*, upper right); however, there were significantly fewer and smaller tumors in livers from DEN-treated Gab2-KO mice (Fig. 2*A*, bottom right). Of note, all WT male mice developed visible hepatic tumor foci after 12 mo of DEN treatment (6 of 6), but only 7 of 14 KO mice presented with a few small tumor nodules (**Table 2**). Statistical analysis indicated that Gab2 deletion dramatically decreased the number, maximum diameter, and volume of liver tumors induced by DEN (Fig. 2*B–D*). Furthermore, the liver weight-to-body weight ratio was employed to evaluate tumor burden in mice. This ratio increased by almost 3-fold in WT mice after DEN treatment but was only mildly increased in Gab2-KO mice (Fig. 2*E*).

**TABLE 2.** Incidence of HCC in mice treated with DEN or saline only

Gab 2 genotype	6 mo	12 mo
DEN treated		
+/+	0/6	6/6
+/−	0/6	7/14
−/−	0/5	7/13
Saline treated		
+/+	0/6	0/7
+/−	0/6	0/8
−/−	0/6	0/12

Ki-67 and phospho-Histone 3 (pH3) are 2 reliable biomarkers of cell proliferation. Here, we analyzed their expression in whole liver tissue by IHC. Few hepatocytes from WT or Gab2-KO mice without DEN treatment were positive for Ki-67 and pH3 (Fig. 2*G, H*, left). Strikingly, the number of Ki-67– and pH3-positive cells increased considerably in liver tumor tissue after DEN treatment (Fig. 2*G, H*, upper right); however, there were fewer Ki-67– and pH3-positive cells in liver tumor tissue from Gab2-KO mice compared with WT mice (Fig. 2*G, H*, lower right). Statistical analysis indicated that Gab2 deletion significantly decreased the number of positive cells for Ki-67 and pH3 (Fig. 2*I, J*), which indicated that Gab2 mediates the growth of hepatic carcinoma.

These observations indicate that knocking out Gab2 significantly inhibits the formation and growth of DEN-induced primary hepatic carcinoma in mice as well as suggest that Gab2 supports hepatic carcinogenesis.

### Overexpression of Gab2 promotes the carcinogenic behavior of hepatocytes

**Figure 3.**
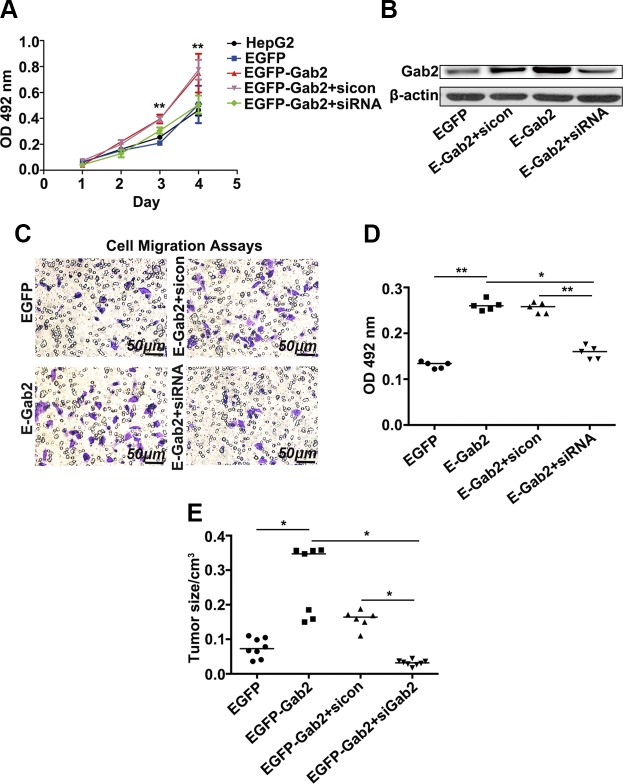
Overexpression of Gab2 increases the tumorigenic activity of HepG2 cells. *A*) Cell growth curves were measured by using MTT assays. *B*) Gab2 protein expression levels in 4 cell lines were determined by Western blotting. *C*, *D*) Representative images of migratory cells in transwell assays after crystal violet staining (*C*, blue color) and quantitative analysis of migratory cells on the basis of staining (*D*). *E*) Statistical analysis of tumor size in nude mice that were injected with HepG2 cells that expressed Gab2. SiCon [control small interfering RNA (siRNA)] or Gab2 siRNA (siRNA). OD, optical density. *P < 0.05, **P < 0.01.

To further explore the mechanism by which Gab2 regulates the carcinogenic behavior of hepatocytes, we constructed HepG2 cell lines that stably express GFP-tagged Gab2 (**Fig. 3*B***). We then investigated the effects of Gab2 on the growth and migration of HCC cells. All cells were cultured for 3 d, and the number of cells was measured daily by using an MTT assay. HepG2 cells have a nice growth curve (Fig. 3*A*, black and blue lines); however, the number of EGFP-Gab2 cells was markedly higher than that of EGFP cells on d 2 and 3 (Fig. 3*A*, red and brown lines), which suggested that Gab2 overexpression enhanced HCC cell growth rate. When intracellular Gab2 was knocked down in these cells by using small interfering RNA, however, the growth rate decreased to the basal level of HepG2 cells (Fig. 3*A*, green line).

Furthermore, we detected the impact of Gab2 on liver cancer cell migration in a migration chamber assay. After incubation for 24 h, some HepG2 cells migrated from the upper surface to the bottom of the chamber (Fig. 3*C*, top left, purple cells); however, EGFP-Gab2 cells were more migratory than EGFP control cells (Fig. 3*C*, bottom left), which indicates that Gab2 overexpression promotes HCC cell migration. As expected, the positive effect of Gab2 on cell migration was abolished by RNA interference–mediated Gab2 knockdown (Fig. 3*C*, bottom right compared with top right). Quantification of migratory cells confirmed the above observation and demonstrated that Gab2 overexpression significantly increased HepG2 cell migration (Fig. 3*D*).

To further evaluate carcinogenic behavior, these cells were injected into nude mice, and tumor formation was monitored. Twenty days after cell inoculation, tumor nodules in the mice were measured. Tumors in mice with Gab2-overexpressing cells were much larger compared with those in control mice, but tumor size was significantly diminished when Gab2 was knocked down in Gab2-overexpressing cells (Fig. 3*E*). These results indicate that Gab2 promotes the development of HCC by enhancing cell proliferation and migration.

### Ablation of Gab2 impairs hepatocellular carcinogenesis

**Figure 4.**
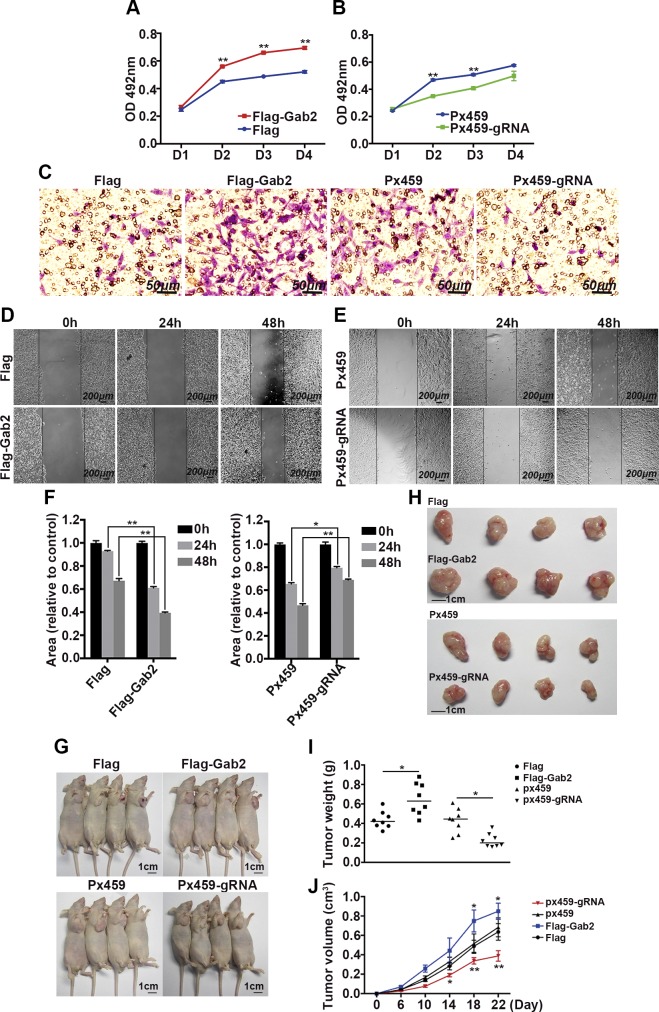
Deletion of Gab2 impairs the tumorigenic activity of HepG2 cells. *A*, *B*) MTT assay for growth of cells with different Gab2 protein levels. *C*) Migration of 4 groups of cell lines in a transwell assay with crystal violet staining. *D*–*F*) Representative images (*D*, *E*) and quantitative analysis (*F*) of the wound healing assay with cells with different Gab2 protein levels. *G*) Representative nude mice that were injected with different cell lines. *H*) Representative tumors from nude mice that were injected with different cell lines. *I*) Tumor weights of the 4 groups of mouse models. *J*) Average tumor growth curves in nude mice. *n* = 8 mice per group. Flag, control for Gab2-overexpressing cells; Flag-Gab2, Gab2-overexpressing cells; Px459, control for Gab2-KO cells; Px459-gRNA, Gab2-KO cells. *P < 0.05, **P < 0.01.

In addition to overexpressing Gab2, we also analyzed the effects of Gab2 deficiency on hepatic carcinogenesis. Gab2 was completely deleted in HepG2 cells by using CRISPR technology. HepG2 cell lines that stably express Flag-tagged Gab2 served as control. MTT assays demonstrated that Gab2 overexpression still promoted liver cancer cell growth, as previously described (**Fig. 4*A***, red line compared with blue line), but Gab2 deletion dramatically restrained HepG2 cell growth (Fig. 4*B*, blue line compared with red line). Transwell migration assay indicated that hepatoma cell migration was enhanced by Gab2 overexpression but inhibited by Gab2 deletion (Fig. 4*C*, purple cells). Moreover, we verified that Gab2 regulates cell migration by using another assay (a wound healing assay; Fig. 4*D–G*). A linear wound was created in confluent cells by using a pipette tip, and the healing rate within the wounded region was evaluated over time. The healing rate of HepG2 cells was accelerated by Gab2 overexpression (Fig. 4*D–E*), but attenuated by Gab2 KO (Fig. 4*F*). In addition, an apoptosis induction experiment also confirmed the critical role of Gab2 on cell viability. Up-regulation of Gab2 promoted, and knockdown of Gab2 repressed, the proportion of apoptotic cells under the treatment of 5-fluorouracil (Supplemental Fig. 2).

Furthermore, these cells were injected into nude mice, and tumor formation was evaluated. From d 6 after injection, tumor size was measured every 4 d for a total of 20 d. At the end of the experiment, tumors were removed from nude mice. Tumors were largest in mice that had been injected with cells that expressed Flag-Gab2 and smallest in mice with Gab2-deleted cells (Px459-gRNA; Fig. 4*G, H*). We also evaluated tumor weights of the 4 groups. As observed, the heaviest weight was measured in the Gab2 overexpression group and the lightest in the Gab2-KO group (Fig. 4*I*). On the basis of the tumor growth curve, overexpression of Gab2 in HepG2 cells markedly increased tumor growth, whereas the deletion of Gab2 in HepG2 cells clearly suppressed tumor development in nude mice (Fig. 4*J*).

These results indicate that Gab2 may promote the initiation and progression of hepatocellular carcinogenesis.

### ERK, Akt, and Jak2 mediate Gab2-induced HepG2 cell proliferation and migration

**Figure 5.**
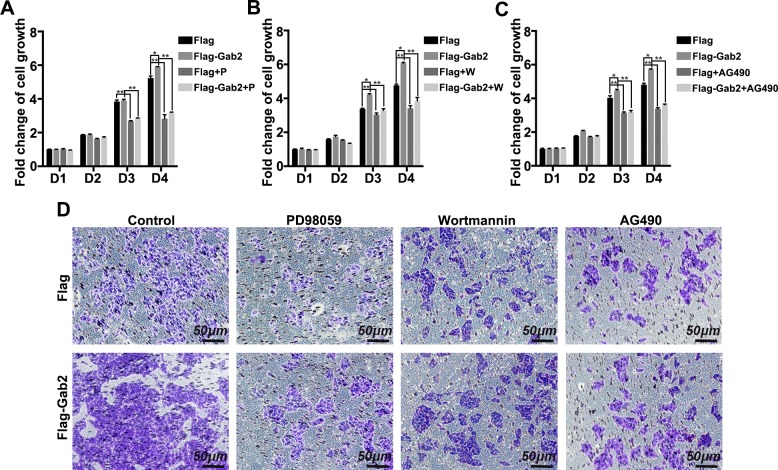
ERK, Akt, and Jak2 inhibitors block Gab2-mediated cell proliferation and metastasis. *A*–*C*) MTT assay of the growth of parental or Gab2-overexpressing cells that were treated or not with PD98059 (P; MEK inhibitor) (*A*), wortmannin (W; PI3K inhibitor) (*B*), or AG490 (Jak2 inhibitor) (*C*). *D*) Transwell assay of the migration of cells that were treated with an inhibitor of MEK, PI3K, or Jak2. D, day. *P < 0.05, **P < 0.01.

Our previous studies have demonstrated that the ERK, PI3K-Akt, and Socs3-Stat3 pathways are involved in the Gab2 regulatory network in fatty liver pathogenesis. To explore the underlying mechanism of Gab2-mediated tumor cell growth and invasion, we determined the influence of the ERK, Akt, and Jak2 pathway inhibitors on the tumor-promoting activity of Gab2. First, HepG2 cell growth was measured with MTT assay. The number of cells that stably express Flag-Gab2 was notably higher than that of controls (Flag-only cell line) after incubation for 3 or 4 d (**Fig. 5*A–C***); however, when cells were pretreated with PD98059 (MEK inhibitor), wortmannin (PI3K inhibitor), or AG490 (Jak2 inhibitor), the number of Flag-Gab2 cells was not obviously increased compared with that of control cells on d 3 and 4 of incubation (Fig. 5*A–C*), which suggested that Gab2 promoted HepG2 cell growth but that pretreatment with an MEK, PI3K, or Jak2 inhibitor inhibited the protumorigenic effects of Gab2. Second, cancer cell migration was examined with a transwell assay. Pretreatment with PD98059, wortmannin, or AG490 notably suppressed Flag-Gab2–mediated cell migration on the basis of the number of transfer cells (Fig. 5*D*). These results indicate that the ERK, Akt, and Jak pathways are potentially involved in the mechanism by which Gab2 regulates hepatic carcinogenesis.

### Gab2 mediates the inflammatory response and inflammatory signaling during hepatic tumorigenesis

**Figure 6.**
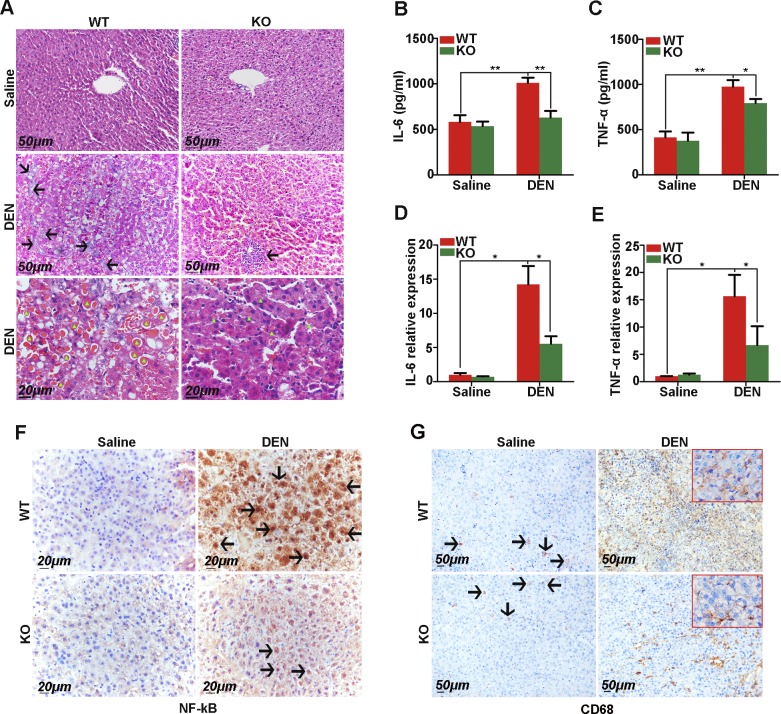
Gab2 deficiency significantly attenuates DEN-induced inflammatory responses in the mouse liver. *A*) Representative images of hematoxylin and eosin–stained tumors in the liver of WT and Gab2-KO mice with or without DEN treatment. Black arrows indicate inflammatory infiltrates. Green triangles indicate eosin-positive cytoplasmic inclusions (red). *B*, *C*) IL-6 (*B*) and TNF-α (*C*) serum levels were measured by ELISA. *D*, *E*) *IL-6* (*D*) and *TNF-*α (*E*) mRNA levels in liver extracts were quantified by quantitative reverse-transcription PCR. *F*) IHC staining of NF-κB in the liver of WT and Gab2-KO mice with saline or DEN treatment. Arrows indicate nucleus activation of NF-κB. *G*) Macrophages were indicated by using IHC staining with CD68-specific Ab. Arrows indicate positive staining of CD68. *P < 0.05, **P < 0.01.

The inflammatory response is a leading inducer of cancer, especially liver cancer. Hepatic inflammation and necrosis can lead to the formation of nodular regenerative hyperplasia in the liver. Here, we observed a marked inflammatory response in liver tissue from DEN-treated mice (**Fig. 6*A***). In the liver tissue from DEN-treated WT mice without a tumor, the trabecular architecture was almost completely lost and was filled with inflammatory infiltrates and eosin-positive cytoplasmic inclusions (Fig. 6*A*, middle and bottom left); however, in DEN-treated Gab2-KO mice, paracarcinoma tissue retained the typical trabecular structure, and only little inflammatory infiltrates and fewer/smaller eosinophilic vacuoles of degeneration were present (Fig. 6*A*, middle and bottom right). Furthermore, we ascertained the levels of the inflammatory chemokines, IL-6 and TNF-α, in serum and paracarcinoma tissues. ELISA analysis demonstrated that Gab2 deletion significantly reduced IL-6 and TNF-α concentrations in serum compared with control (DEN-treated WT animals; Fig. 6*B, C*). Quantitative PCR results revealed that knocking out Gab2 inhibited IL-6 and TNF-α transcription in paracarcinoma tissues (Fig. 6*D, E*). In addition, we also performed IHC staining with inflammatory cell markers to support our findings. As indicated in Fig. 6*F*, NF-κB protein expression demonstrated low expression and was localized in the cytoplasm in saline-treated WT and Gab2-KO mice; however, under the stimulation of DEN, NF-κB was activated after being freed up to enter the nucleus in mice liver tissue and there was more nucleus activation of NF-κB in DEN-treated WT mice than in the DEN-treated Gab2-KO group (Fig. 6*F*, right). For staining of CD68, a cell surface marker of macrophages, we also observed that CD68^+^ cells were robustly increased with treatment with DEN compared with the saline-treated group; however, there were obviously a smaller number of CD68^+^ cells in the Gab2-KO group than in the WT group that was treated with DEN (Fig. 6*G*, right). These results indicate that knocking out Gab2 suppresses inflammatory lesions, cytokine production, and inflammatory cell formation in hepatic carcinogenesis.

**Figure 7.**
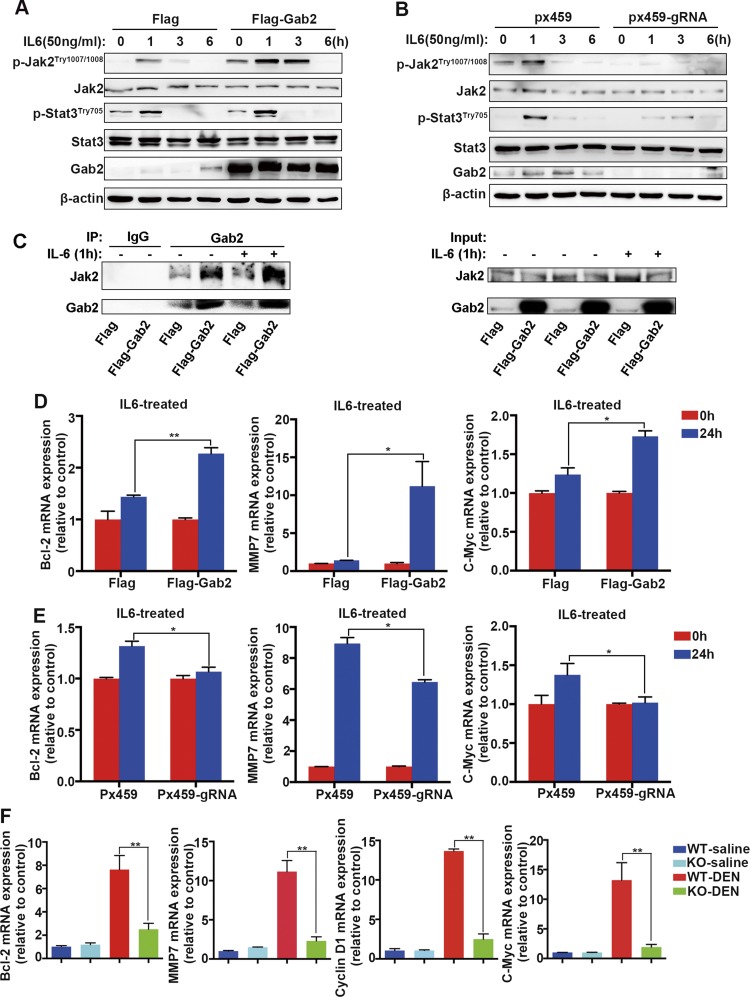
Gab2 modulates the IL-6 signaling pathway and activates the expression of downstream target genes. *A*, *B*) IL-6 induction of Jak2 and Stat3 phosphorylation in HepG2 cells with or without Gab2 protein was analyzed by Western blotting with p-Stat3^Tyr705^ and p-Jak2^Tyr1007/1008^ Abs. β-Actin was used as loading control. *C*) Interaction between Gab2 and Jak2 was determined by coimmunoprecipitation in Flag and Flag-Gab2 group cells with or without IL-6 treatment. *D*) mRNA levels of *c-Myc*, *Bcl-2*, and *MMP7* (matrix metalloproteinase-7), downstream genes in the IL-6 signaling pathway in HepG2 cells that overexpress Gab2. *E*) *c-Myc*, *Bcl-2*, and *MMP7* gene expression in Px459 and Px459-gRNA cell lines after IL-6 treatment for 24 h. *F*) mRNA levels of *c-Myc*, *Bcl-2*, *MMP7*, and *cyclin D1* in whole liver extracts. Flag, control for Gab2-overexpressing cells; Flag-Gab2, Gab2-overexpressing cells; Px459, control for Gab2-KO cells; Px459-gRNA, Gab2-KO cells. *P < 0.05, **P < 0.01.

To define the role of Gab2 in hepatic inflammatory signal transduction, we investigated the effect of Gab2 on the IL-6 signaling pathway. HepG2 cells were cultured without serum for 12 h, then treated with 50 ng/ml IL-6 for 1, 3, and 6 h. In control cells, Jak2 and Stat3 phosphorylation was observed at 1 h, but disappeared by 3 h after IL-6 stimulation (**Fig. 7*A***, left 4 columns); however, in Gab2-overexpressing cells, IL-6–induced phosphorylation of Jak2 and Stat3 was significantly increased at 1 h, and Jak2 phosphorylation remained high at 3 h after IL-6 induction (Fig. 7*A*, 6th column in the 1st and 3rd top panels). Conversely, IL-6–induced phosphorylation of Jak2 and Stat3 was remarkably decreased when Gab2 was knocked out in HepG2 cells (Fig. 7*B*, 6th column compared with 2nd column in the 1st and 3rd top panels). Of interest, we observed moderate up-regulation of Gab2 in empty vector transfected cells after IL-6 treatment (Fig. 7*A, B*, left 4 columns). It is possible that this reflects an up-regulation of endogenous Gab2 in response to IL-6, which indicates that Gab2 might directly mediate IL-6 signaling by regulating Jak2 and Stat3 phosphorylation in hepatocarcinoma cells. Of importance, we found that Jak2 was a key interactant of Gab2 in hepatoma cells. Without stimulation, forced expression of Gab2 robustly enhanced the binding of Jak2 to Gab2. IL-6 treatment could further strengthen the interaction between Jak2 and Gab2 in Gab2-overexpressing cells (Fig. 7*C*). These findings indicate that Gab2 modulates IL-6–mediated the Jak2/Stat3 signaling pathway, potentially *via* interaction with Jak2.

To further understand the effect of Gab2 on IL-6 signaling, we analyzed mRNA levels of downstream target genes. Gab2 overexpression enhanced the IL-6–stimulated expression of *Bcl-2* (B-cell lymphoma 2), *c-Myc*, and *MMP7* (matrix metalloproteinase-7), which are involved in cell growth, apoptosis, survival, and migration induced by IL-6 (Fig. 7*D*). As expected, Gab2 ablation significantly inhibited the IL-6–stimulated expression of these genes (Fig. 7*E*). Consistently, we also observed an increased expression of the *Bcl-2*, *c-Myc*, *cyclinD1*, and *MMP7* target genes in primary liver cancer tissue from DEN-treated mice (Fig. 7*F*, red columns); however, the DEN-stimulated expression of these genes was significantly restrained by the absence of Gab2 (Fig. 7*F*, green columns). These results suggest that Gab2 protein levels affect the expression of certain downstream target genes that are regulated by Stat3 and also confirm that Gab2 is involved in the IL-6–induced Jak2/Stat3 signaling pathway. On the basis of these observations, Gab2 might participate in IL-6–induced signaling during hepatic inflammatory responses.

## DISCUSSION

Hepatocellular carcinogenesis is an extremely elusive and complex process that involves numerous intricate mechanisms, which greatly hampers the development of effective therapies for HCC (25). The present study defines the oncogenic regulation by Gab2 by using animal models and stable cell lines and provides powerful evidence that supports the use of Gab2 as a potential therapeutic target for the treatment of HCC.

In this study, we evaluated Gab2 expression in fresh HCC tissue and tissue microarrays and found that Gab2 was overexpressed in ∼60 to 70% of HCC samples, but had low expression in paracarcinoma or normal liver tissue. These findings indicate that Gab2 might be a tumor-associated protein. Two recent studies investigated Gab2 expression in HCC samples and confirm our results (26, 27). Gab2 is also associated with tumor behavior in other types of malignancies, such as breast cancer (28), melanoma (29), ovarian cancer (30), lung cancer (31), glioma (32), and gastric cancer (33). For instance, 57% of estrogen receptor-positive breast cancer specimens have high Gab2 expression compared with normal breast tissue (34). Increased Gab2 DNA copy number and gene amplification are present in a subset of melanoma samples (35). In addition, Gab2 mRNA and protein expression is notably up-regulated in ovarian serous cystadenocarcinoma—a subtype of ovarian adenocarcinoma with poor survival—compared with normal ovarian tissue (36, 37).

Furthermore, we defined the oncogenic role of Gab2 by using transgenic mouse models and HCC cell lines. Deletion of Gab2 blocked DEN-induced primary liver cancer in mice. Overexpression of Gab2 in HCC cells promoted proliferation, migration, and xenograft tumor growth in nude mice, whereas Gab2 deficiency impaired these oncogenic effects in HepG2 cells. Of interest, oncogenic regulation by Gab2 also occurs in other types of tumors. Up-regulation of Gab2 in MCF-10A cells contributes to increased proliferation and metastasis in a manner that is dependent on EGF and other growth factors (28, 34, 38). Forced expression of Gab2 can promote the proliferation and invasion of melanoma and breast cancer cells (35, 39). Overexpression of Gab2 promotes tumor angiogenesis by increasing the expression of multiple chemokines in ovarian cancer (40), and Gab2 also promotes the growth of cancer cells in lung cancer (41) and glioma (42).

Gab2 is a signal docking protein that recruits several intracellular signaling pathways. In carcinogenesis, Gab2 engages various signaling pathways in different types of cancer. Gab2 can recruit Shp2 to activate Ras/ERK signaling in breast cancer and melanoma (43, 44), and the PI3K/Akt signaling pathway has been reported to be modulated by Gab2 in breast cancer (45), ovarian cancer (30, 37), and melanoma (46). Gab2 is also required for Bcr-Abl–mediated myeloid and lymphoid transformation by affecting the Shp2/Stat5 and Jak/Stat signaling networks (47–50). Moreover, Gab2 contributes to the angiogenic switch by increasing hypoxia-inducible factor-1α and VEGF levels in melanoma (29). In glioblastoma, both miRNA-197 and miR125a-5p inhibit glioma cell proliferation and invasion by negatively regulating Gab2 (51, 52). In the present study, inhibitors of PI3K, MEK, or Jak2 significantly suppressed Gab2-mediated HepG2 cell proliferation and migration. In addition, our previous study demonstrated that Gab2 could interact with PI3K, Socs3, and Shp2 in HepG2 cells upon treatment with ethanol or oleic acid, which were also the carcinogenic factors of liver cancer (22). These results thus suggest that Gab2 integrates multiple signaling molecules to regulate the development of HCC.

Inflammation is a main inducer of cancer, especially liver cancer. A high percentage of HCC cases derive from hepatitis. In previous studies, we demonstrated that Gab2 mediates steatohepatitis. Here, the ablation of Gab2 attenuated the inflammatory response in DEN-induced primary liver cancer in mice and decreased the production of inflammatory cytokines and the quantity of macrophages. Thus, Gab2 mediates the inflammatory response in liver tissue and is involved in the development of hepatitis-induced liver cancer. Furthermore, we evaluated the effects of Gab2 on signal transduction by inflammatory cytokines and found that Gab2 mediated IL-6–activated Jak2/Stat3 signaling and regulated the expression of downstream IL-6 target genes. In addition, the Jak2 inhibitor, AG490, markedly restrained the ability of Gab2 to promote hepatoma cell proliferation and metastasis. IL-6 is a typical inflammatory cytokine that stimulates inflammation and autoimmune responses in many diseases, including diabetes (53), Alzheimer’s disease (54), and some tumors (55, 56). The Jak/Stat pathway is a key signaling pathway that transduces signals from inflammatory factors, including such inflammatory cytokines as IL-6, to modulate the inflammatory response. Most studies have confirmed that aberrant IL-6–Jak/Stat signaling facilitates HCC progression (57, 58). Our results demonstrate that Gab2 mediates the inflammatory response induced by IL-6 in hepatocellular carcinogenesis and shed light on the mechanism by which Gab2 regulates inflammatory signaling.

In summary, this study demonstrated that Gab2 is a cancer-associated protein that plays an oncogenic role in hepatocellular carcinogenesis by integrating multiple signaling pathways, especially those that are modulated by inflammatory factors; therefore, the signaling adaptor protein, Gab2, may be a powerful, novel target for the prevention and treatment of liver cancer.

## Supplementary Material

Supplemental Data

## Abbreviations

Bcl-2: B-cell lymphoma 2
DEN: diethylnitrosamine
Gab2: growth factor receptor-bound protein 2–associated binding protein 2
HBV: hepatitis B virus
HCC: hepatocellular carcinogenesis
IHC: immunohistochemistry
Jak: Janus kinase
KO: knockout
MTT: 3-(4,5-dimethylthiazol-2-*yl*)-2,5-diphenyltetrazolium bromide
pH3: phospho-histone 3
Socs3: suppressor of cytokine signaling 3
Stat3: signal transducer and activator of transcription 3
WT: wild type
