# Supplementary material for: Gab2 mediates hepatocellular carcinogenesis by integrating multiple signaling pathways
Source: FASEB J. 2017 Aug 21;31(12):5530–42. doi: 10.1096/fj.201700120RR (PMC5690380; doi:10.1096/fj.201700120RR)
Supplement: Supplemental Data [file supp_31_12_5530__index.html]

Gab2 mediates hepatocellular carcinogenesis by integrating multiple signaling pathways — Gab2 mediates hepatocellular carcinogenesis by integrating multiple signaling pathways — Supplemental Data 

# Gab2 mediates hepatocellular carcinogenesis by integrating multiple signaling pathways

## Supplemental Data

- Supplemental Data
