# Supplementary material for: Gab2 mediates hepatocellular carcinogenesis by integrating multiple signaling pathways
Source: FASEB J. 2017 Aug 21;31(12):5530–42. doi: 10.1096/fj.201700120RR (PMC5690380; doi:10.1096/fj.201700120RR)
Supplement: Supplemental Data [file supp_fj.201700120RR_Supplemental_Data.pdf]

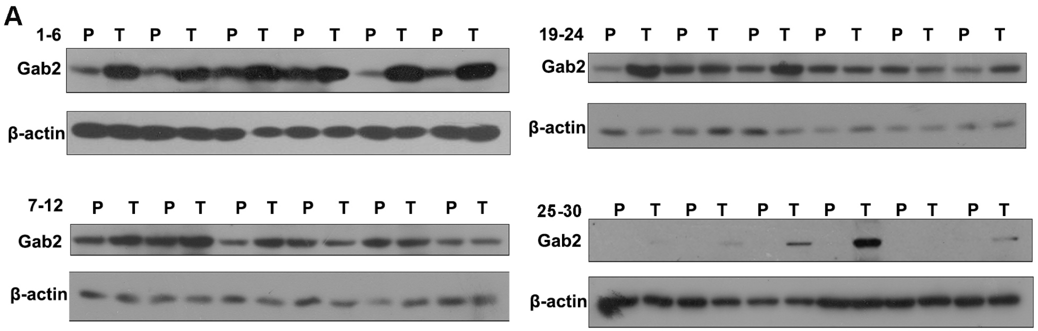

Supplementary Figure 1

Supplementary Figure 1 Gab2 protein expression was up-regulated in HCC samples. (A) The expression of Gab2 was detected in 30 HCC patients by western blotting. P, para-carcinoma. T, Tumor.

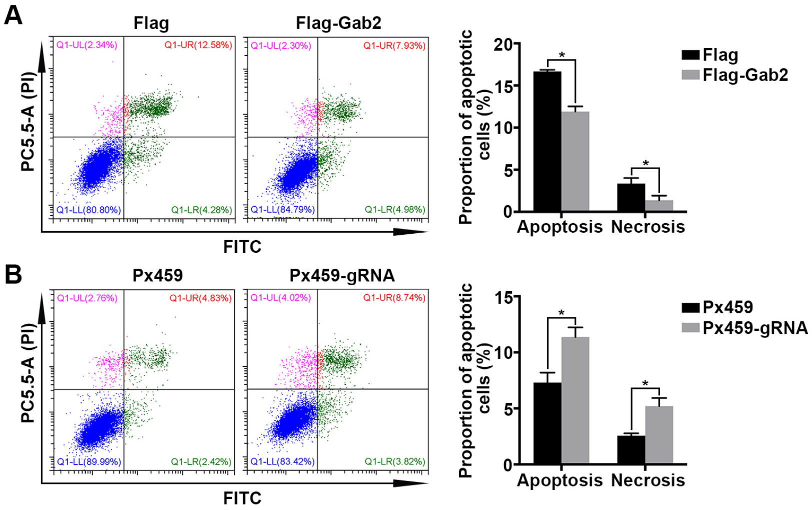

Supplementary Figure 2

Supplementary Figure 2 Effects of Gab2 on cell apoptosis of HepG2. (A) The number and proportion of apoptotic cells in Flag and Flag-Gab2 group cells. **(B)** The number and proportion of apoptotic cells in Px459 and Px459-gRNA group cells. * P<0.05.

Supplementary Table 1. Description of primers used for qRT-PCR

Gene	Sense primer(5′-3′)	Antisense primer(5′-3′)
Human		
c-Myc	5′-GGCTCCTGGCAAAAGGTCA-3′	5′-CTGCGTAGTTGTGCTGATGT-3′
Bcl-2	5′-GGTGGGGTCATGTGTGTGG-3′	5′-CGGTTCAGGTACTCAGTCATCC-3′
MMP7	5′-GAGTGAGCTACAGTGGGAACA-3′	5′-CTATGACGCGGGAGTTTAACAT-3′
Gapdh	5′-GGAGCGAGATCCCTCCAAAAT-3′	5′-GGCTGTTGTCATACTTCTCATGG-3′
Mouse		
c-Myc	5′-CCCTATTTCATCTGCGACGAG-3′	5′-GAGAAGGACGTAGCGACCG-3′
Bcl-2	5′-GCTACCGTCGTGACTTCGC-3′	5′-CCCCACCGAACTCAAAGAAGG-3′
MMP7	5′-TCGCAAGGAGAGATCATGGAG-3′	5′-CTGCGTCCTCACCATCAGTC-3′
CyclinD1	5′-TGACTGCCGAGAAGTTGTGC-3′	5′-CTCATCCGCCTCTGGCATT-3′
Gapdh	5′-GGTGAAGGTCGGTGTGAACG-3′	5′-CTCGCTCCTGGAAGATGGTG-3′
